# Bilateral pontine infarction with basilar artery fenestration: A case report

**DOI:** 10.1097/MD.0000000000021530

**Published:** 2020-08-07

**Authors:** Sang Hee Ha, Hyug-Gi Kim, Bum Joon Kim

**Affiliations:** a Department of Neurology; b Department of Radiology, Kyung Hee University Hospital; c Department of Neurology, Asan Medical Center, Seoul, Republic of Korea.

**Keywords:** basilar artery fenestration, bilateral pontine infarction, hemodynamics

## Abstract

**Rationale:**

Basilar artery (BA) fenestration is a congenital anomaly with duplicated BA, which can cause ischemic stroke. However, the stroke mechanism is not clearly verified in patients with BA fenestration.

**Patient concerns:**

Here, we report a case of 64-year-old man with well-controlled hypertension admitted with dysarthria, only.

**Diagnoses:**

Diffusion weighted image showed a bilateral symmetric pontine infarction sparing the midline. BA fenestration was observed from magnetic resonance angiography.

**Intervention:**

High-resolution magnetic resonance image (MRI) and 4D flow MRI was performed to verify the mechanism of stroke associated with BA fenestration.

**Outcomes:**

No plaque was observed at the area of BA fenestration from high-resolution MRI. 4D flow MRI showed bifurcated flow with high flow velocity and low shear stress at the area of BA fenestration.

**Lessons:**

A turbulent flow with high flow velocity and low shear stress at the BA fenestration area may have influenced the flow through the bilateral perforating arteries resulting in a bilateral symmetric pontine infarction with sparing the midline where the septa of BA is located. 4D flow dynamic studies may be beneficial for verifying the mechanism of stroke.

## 1 Introduction

Fenestration of basilar artery (BA) is a rare but well-described congenital anomaly. BA is duplicated, especially in the lower end, because of the failure of plexiform primitive longitudinal neural artery fusion. Previously, several cases of posterior circulation infarction or cerebral aneurysms due to BA fenestration were reported.^[1]^ However, the mechanism involved in posterior circulation infarction in patients with BA fenestration was not clearly verified. Here we report of case of patient with symmetrical bilateral pontine infarction patient due to BA fenestration, which may help understanding the mechanism of ischemic stroke. Informed written consent was obtained from the patient for publication of this case report and accompanying images. As a case report ethical approval of this study was waived by the local ethics committee.

## 2 Case report

**Figure 1:**
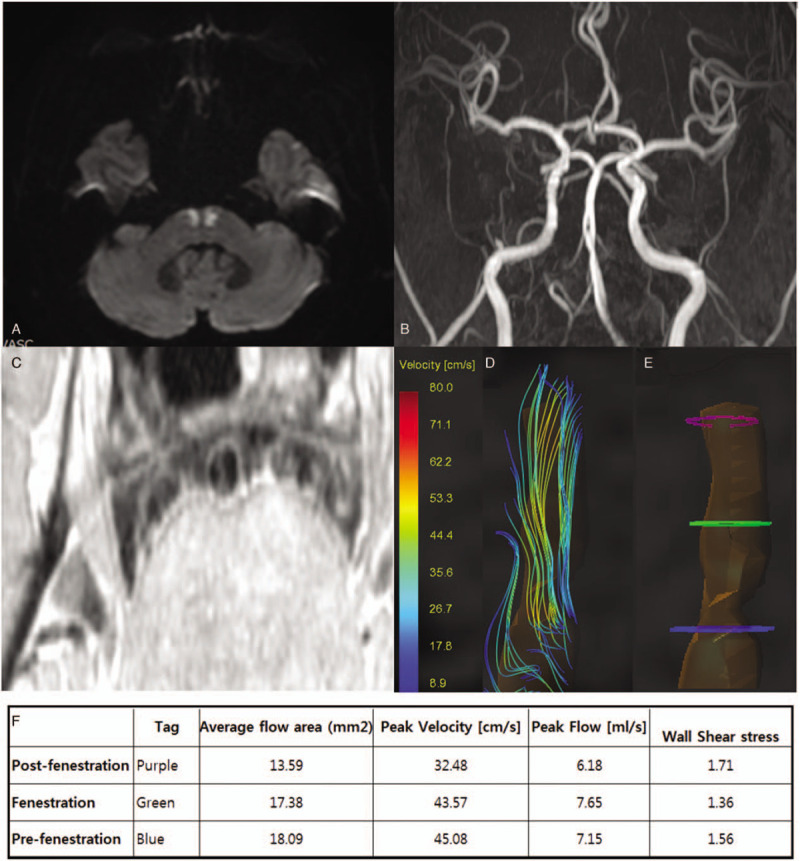
Bilateral pontine infarction with basilar artery fenestration. Axial diffusion-weighted image MRI (A) with symmetric bilateral pontine infarction sparing the midline of basis pontis and time-of-flight magnetic resonance angiography (B) show basilar artery fenestration. High-resolution MRI (C) reveal intraluminal wall and dual lumen in the basilar artery. Basilar artery flow trajectory presented at systolic (D) period demonstrating bifurcated blood flow. Hemodynamic parameters measured from pre-fenestration (blue), fenestration (green), and post-fenestration (purple) area (E) shows low wall shear stress at the fenestration area (F).

A 64-year-old man suddenly developed severe dysarthria 2 days before admission. Neurological examination revealed no other deficit. There was no risk factor for stroke, except hypertension. The patient was under regular medication of calcium channel blocker and his blood pressure was well-controlled. Diffusion-weighted image revealed symmetric high-signal intensity in bilateral paramedian pontine (Fig. 1A). The midline of basis pontis was spared. The magnetic resonance angiography displayed fenestration of the proximal mid-BA (Fig. 1B). High-resolution magnetic resonance image (HRMRI) showed endothelium-lined partial intraluminal septa within the fenestrated artery at the level of bilateral symmetric pontine infarction (Fig. 1C). No atheroma was observed from HRMRI. Flow dynamic study with GTflow (GyroTools, Winterthur, Switzerland) was performed. Flow was divided at the fenestrated area (Fig. 1D). Flow velocity and wall shear stress was measured at before, after, and at the level of fenestration (Fig. 1E). Among those 3 points, peak flow velocity was highest and the wall shear stress was lowest at the area of fenestration (Fig. 1F). Aspirin and cilostazol were started and 3 months later patient was maintained with cilostazol mono therapy. Calcium channel blocker was reused after the acute period of stroke. Blood pressure was controlled under 130/80 mmHg. Dysarthria improved and no stroke recurrence was observed during the follow-up.

## 3 Discussion

Previously, pediatric stroke patients with BA fenestration, but without vascular risk factors were reported.^[1]^ Therefore, fenestration of BA itself may be the direct cause of ischemic stroke. However, the exact mechanism was not verified.

It is well-known that local hemodynamics alters the development of atherosclerosis; at the bifurcated area of carotid artery, the turbulence at the outer wall influences the development of atherosclerosis.^[2]^ Similarly in BA fenestration, HRMRI study revealed that plaques were mostly observe at the lateral walls, which may obliterate the orifice of BA perforators, in the fenestrated BA area.^[3]^ The location of plaques were explained by the turbulence and low shear stress at the area.

However, our case did not show a plaque from the HRMRI. Still an area with turbulence may exist and cause low flow through the perforator originating at the area with turbulence.^[4]^ Interestingly, our patients showed a very symmetric lesion sparring the midline where the septa was observed, which implies that hemodynamic factor may have bilaterally influenced the occurrence of symmetric bilateral pontine infarction. The flow parameters measured at the area of BA fenestration was also supportive for the hemodynamic hypothesis showing that the peak systolic velocity was highest and the wall shear stress was lowest at the fenestrated area, where the bilateral symmetric infarction was observed.^[5]^ Area with turbulence is characterized by high flow velocity and low shear stress.

Our case helps to explain the mechanism of pontine infarction in patients with BA fenestration without vascular risk factor by a hemodynamic theory. Furthermore, HRMRI and 4D flow studies may be helpful for evaluating the stroke mechanism.

## Author contributions

SH Ha contributed by making the concept and the first draft of the manuscript.

HG Kim contributed by analyzing the imaging data and revising the manuscript.

BJ Kim contributed by making the concept analyzing the data and revising the manuscript.
